# Malaria vectors in South America: current and future scenarios

**DOI:** 10.1186/s13071-015-1038-4

**Published:** 2015-08-19

**Authors:** Gabriel Zorello Laporta, Yvonne-Marie Linton, Richard C. Wilkerson, Eduardo Sterlino Bergo, Sandra Sayuri Nagaki, Denise Cristina Sant’Ana, Maria Anice Mureb Sallum

**Affiliations:** Departamento de Epidemiologia, Faculdade de Saúde Pública, Universidade de São Paulo, São Paulo, SP Brazil; Laboratório de Informática Médica, Faculdade de Medicina, Universidade de São Paulo, São Paulo, SP Brazil; Setor de Pós-graduação, Pesquisa e Inovação, Faculdade de Medicina do ABC, Santo André, SP Brazil; Division of Entomology, Walter Reed Army Institute of Research, Silver Spring, MD USA; Walter Reed Biosystematics Unit, Museum Support Center, Smithsonian Institution, Suitland, MD USA; Department of Entomology, National Museum of Natural History, Smithsonian Institution, Washington, DC USA; Department of Preventative Medicine and Biostatistics, Uniformed Services University of the Health Sciences, Bethesda, MD USA; Superintendência de Controle de Endemias (SUCEN), Secretaria de Estado da Saúde de São Paulo, Araraquara, SP Brazil

**Keywords:** *Anopheles darlingi*, Albitarsis Complex, Climate change, Ecological niche model, *Plasmodium falciparum*

## Abstract

**Background:**

Malaria remains a significant public health issue in South America. Future climate change may influence the distribution of the disease, which is dependent on the distribution of those *Anopheles* mosquitoes competent to transmit *Plasmodium falciparum*. Herein, predictive niche models of the habitat suitability for *P. falciparum*, the current primary vector *Anopheles darlingi* and nine other known and/or potential vector species of the Neotropical Albitarsis Complex, were used to document the current situation and project future scenarios under climate changes in South America in 2070.

**Methods:**

To build each ecological niche model, we employed topography, climate and biome, and the currently defined distribution of *P. falciparum*, *An. darlingi* and nine species comprising the Albitarsis Complex in South America. Current and future (i.e., 2070) distributions were forecast by projecting the fitted ecological niche model onto the current environmental situation and two scenarios of simulated climate change. Statistical analyses were performed between the parasite and each vector in both the present and future scenarios to address potential vector roles in the dynamics of malaria transmission.

**Results:**

Current distributions of malaria vector species were associated with that of *P. falciparum*, confirming their role in transmission, especially *An. darlingi*, *An. marajoara* and *An. deaneorum*. Projected climate changes included higher temperatures, lower water availability and biome modifications. Regardless of future scenarios considered, the geographic distribution of *P. falciparum* was exacerbated in 2070 South America, with the distribution of the pathogen covering 35-46 % of the continent. As the current primary vector *An. darlingi* showed low tolerance for drier environments, the projected climate change would significantly reduce suitable habitat, impacting both its distribution and abundance*.* Conversely, climate generalist members of the Albitarsis Complex showed significant spatial and temporal expansion potential in 2070, and we conclude these species will become more important in the dynamics of malaria transmission in South America.

**Conclusions:**

Our data suggest that climate and landscape effects will elevate the importance of members of the Albitarsis Complex in malaria transmission in South America in 2070, highlighting the need for further studies addressing the bionomics, ecology and behaviours of the species comprising the Albitarsis Complex.

**Electronic supplementary material:**

The online version of this article (doi:10.1186/s13071-015-1038-4) contains supplementary material, which is available to authorized users.

## Background

Current consensus among scientists is that global patterns of climate warming observed over the past twenty years are attributable to human activities [1–3], and public statements endorsing this position have been issued by scientific organizations worldwide [4, 5]. Some health-related issues of humans and animals are correlated to climate change, including heat-related disorders, respiratory disorders, vector-borne diseases, water-borne diseases, food insecurity, and mental health disorders that are associated with natural disasters [6].

The epidemiology of vector-borne diseases has been under the influence of climate change at the global level since the last quarter of the 20th Century [7]. Consequently, the impact of climate factors on malaria reemergence or emergence has attracted considerable attention in studies relating climate factors to mosquito ecology [8, 9]. This particular interest reflects the fact that malaria is a significant public health burden and, concomitantly, the dynamics of transmission are highly sensitive to environmental conditions [10, 11]. Additionally, the future distribution of malaria is dependent on the distribution of competent *Anopheles* vectors, especially those that are exophagic climate generalists [12].

In Brazil, the number of malaria cases attributable to *P. falciparum* is decreasing annually, mainly because of the successful control and elimination strategies adopted by the Malaria Control Program [13]. In 2012, *P. falciparum* accounted for only 10 % out of 276,000 confirmed malaria cases [14]. *Anopheles darlingi* Root is the primary malaria vector in South America, transmitting both *P. falciparum* and *P. vivax* in Brazil [15, 16], but many other species are also involved in the dynamics of the transmission of *Plasmodium* [17]. These include members of the Albitarsis Complex that have attracted attention because of the high number of new species described in recent years [18–22]. The Albitarsis Complex comprises five formally described species (*An. albitarsis* Lynch Arribalzaga, *An. deaneorum* Rosa-Freitas, *An. janconnae* Wilkerson and Sallum, *An. marajoara* Galvao and Damasceno, *An. oryzalimnetes* Wilkerson and Motoki), and three recognized unnamed species, *An. albitarsis* F [20], *An. albitarsis* G [21, 22], *An. albitarsis* I [22] [20–24]. *Anopheles albitarsis* H was originally conservatively described as a mitochondrial lineage due to low sample size [22], but has since been more widely detected and should be considered a separate species (Linton & Wilkerson, pers. comm.). Herein all nine taxa are treated as separate species. *Anopheles deaneorum*, *An. marajoara*, and *An. janconnae* are proven vectors of *Plasmodium* in Brazil [22, 25–27]. Areas currently predicted as suitable for these species (plus *An. albitarsis* G, *An. albitarsis* H, and *An. albitarsis* I) largely coincide with distribution models of *P. falciparum* and *P. vivax* [24].

The Amazonian region has been identified as an especially vulnerable area for future climate changes because of the projected increase in the length of the dry season [28, 29], which will impact on the dynamics of infectious diseases [30]. The stability and resilience of the Amazonian climate-vegetation dynamics has been the focus of many studies aiming to understand the impacts of climate change on forest dynamics, and its potential long-term replacement by drier biomes such as the tropical savanna [31, 32], as during the mid-Pliocene [33]. Within this context, a tropical savanna biome would be more favourable for the Albitarsis Complex [24, 26]. This group is mainly associated with *Plasmodium* transmission as secondary vectors [24]. However, *An. marajoara* can act as a primary vector depending on the ecological changes, especially those related to land-use and human migration [26]. Therefore, it is important to address the effects of a potential savannization of the Amazon forest on the dynamics of malaria transmission.

This study employed habitat suitability niche modelling to address potential associations among the spatial distribution of *P. falciparum*, *An. darlingi* and the nine component species of the Albitarsis Complex, considering the current environmental scenario and two scenarios of climate changes predicted for the year of 2070. The objectives of the study were to: 1) model the potential ecological niche of *P. falciparum*, nine species in the Albitarsis Complex and *An. darlingi*; 2) estimate potential spatial distribution of nine species in the Albitarsis Complex, *An. darlingi* and *P. falciparum* in two distinct scenarios of climate changes predicted for the year of 2070; and 3) identify potential associations between the spatial distribution of *P. falciparum* and nine Albitarsis Complex species and *An. darlingi*.

## Methods

### Specimen data used for analysis

The number of occurrences of *P. falciparum* and each *Anopheles* species, and absence data, utilized in the species distribution modelling are shown in Table 1.

**Table 1:** Number of occurrences (presence data) of *P. falciparum* and each *Anopheles* species and absence data utilized in the species distribution modelling approach with the aid of the MaxEnt algorithm and Boosted Regression Trees

	MaxEnt		Boosted Regression Trees		
	Presence data	Absence data (background points, pseudo-absence)	Presence data (train data)	Presence data (test data)	Absence data (derived from MaxEnt output)
*P. falciparum*	112	200	84	28	10
*An. darlingi*	66	200	50	16	10
*An. albitarsis s.s.*	138	200	104	34	10
*An. oryzalimnetes*	240	200	180	60	10
*An. marajoara*	153	200	115	38	10
*An. deaneorum*	70	200	53	17	10
*An. janconnae*	96	200	72	24	10
*An. albitarsis *F	44	200	33	11	10
*An. albitarsis *G	106	200	80	26	10
*An. albitarsis *H	88	200	66	22	10
*An. albitarsis *I	12	200	9	3	10

**Fig. 1:**
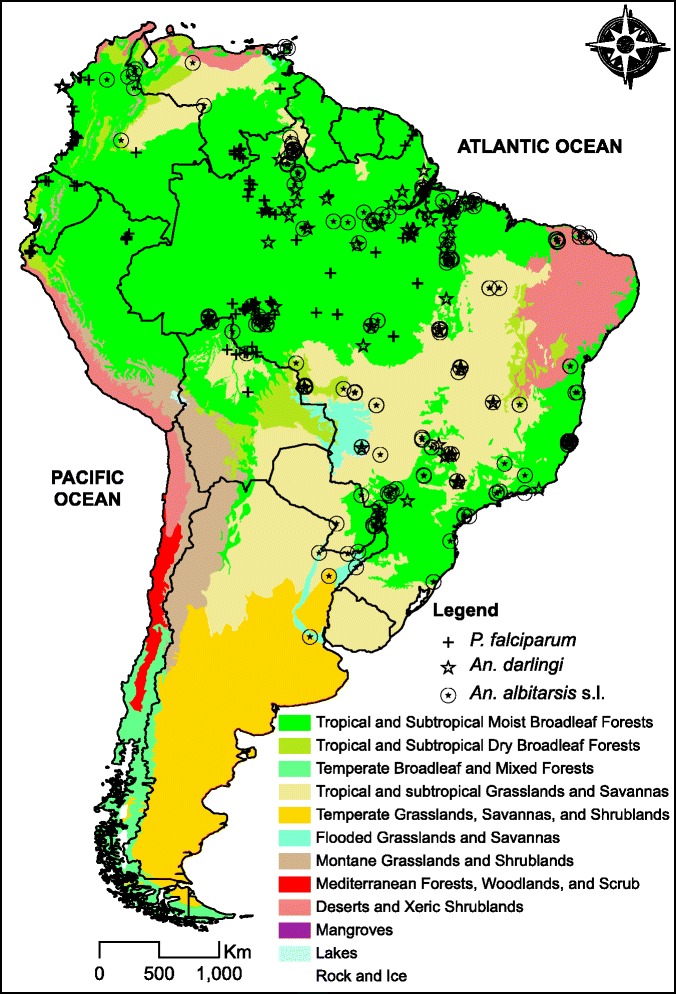
Contemporary terrestrial biomes and occurrences of *P. falciparum*, *An. darlingi* and nine species in the Albitarsis Complex in South America. Sources: Biomes (the World Wildlife Fund), *P. falciparum* (the Malaria Atlas Project), *An. darlingi* (published data [27, 34, 35], plus new data obtained by ESB/MAMS [Additional file 1]) and the Albitarsis Complex (published data [22, 24]). Projection: longitude-latitude. Datum: WGS84

The distributions of *Anopheles* species and *P. falciparum* are shown in Fig. 1.

The geographical distribution of *An. darlingi* was obtained from the published literature [27, 34, 35] and new data was obtained from field collections by ESB and MAMS (Additional file 1). Specimens of adult *An. darlingi* were identified by morphology using available identification keys [36].

The distribution of *P. falciparum* was obtained from The Malaria Atlas Project (MAP) [37, 38].

Species level distribution data for members of the Albitarsis Complex was achieved through molecular species verification of 1131 specimens, including topotypic and type series material, using DNA barcode sequences (658 bp of the mitochondrial cytochrome c oxidase I gene) (see [22] for methodology). These data, generated by this team, have been previously published under the following GenBank Accession numbers: JQ614998 - JQ615562 [22], KJ011904 - KJ012004, KJ492398 - KJ492558 and KJ492676 - KJ492894 [24]. Detailed individual specimen level collection data are available in the Mosquito Barcoding Initiative projects on the Barcode of Life (BOLD) [39] website under the Mosquitoes of the World container project, “MBIAA: *Anopheles albitarsis* complex”, and are available for download on VectorMap [40].

Template DNAs corresponding to GenBank Accession numbers KJ492676 - KJ492894 (n = 219) [24] collected by MAMS and ESB are retained at −70 °C in an entomological reference collection, the Coleção Entomológica de Referência of the Universidade de São Paulo, SP Brasil. Template DNA and other associated life stages for the remaining 912 specimens collected by RCW corresponding to GenBank Accession numbers JQ614998 - JQ615562 [22], KJ011904 - KJ012004 and KJ492398 - KJ492558 [24] are housed at the Smithsonian Institution National Museum of Natural History (NMNH), USA.

### Environmental variables

Bioclimatic, topographic and biome variables were used from different data sources. The bioclimatic data were obtained from the WorldClim [41], the elevation data were from the Shuttle Radar Topography Mission (SRTM) [42] and the terrestrial biomes were from the World Wildlife Fund (WWF) [43] (see Additional file 2, Additional file 3).

### Species distribution modelling

The spatial relationship between the environmental variables and presence of each *Anopheles* species and *P. falciparum* were carried out using the MaxEnt algorithm and Boosted Regression Trees (BRT) (see Table 1 for more information). Implementations of these methods are in the software R 3.0.1, in the following packages: *dismo*, *gbm*, *raster*, *rgdal* and *rJava* [44–48]. Further information about these methods, applied for species distribution modelling, can be found elsewhere, e.g., MaxEnt [49] and BRT [50]. For the predicted presence of *P. falciparum,* it was herein assumed that the presence of the parasite is related to environmental factors. This was considered fundamental for any climate scenario because the mosquito host is an ectotherm, and therefore the dynamics of the transmission suffers the influence of climate variables, such as temperature and precipitation (e.g., [8, 10]). Calculation of the average value between the two methods, MaxEnt and BRT, was performed in order to produce a consensus species distribution model.

### Environmental variables in future scenarios

The WorldClim [51] provides bioclimatic variables from global climate models by the National Aeronautics and Space Administration (NASA) [52] and the European Network for Earth System Modelling (ENES) [53] in the fifth assessment of the Intergovernmental Panel on Climate Change, under the representative concentration pathway RCP85 (see Additional file 4, Additional file 5, and Additional file 6 for more information). The rationale for using the worst scenario among the four possible representative concentration pathways was that it would yield predictions for the most pessimistic scenario considered by the IPCC-CMIP5. The most pessimistic scenario represented a situation in which human behaviours related to climate change would stay unchanged between current and future scenarios. For the simulation tests, we assumed that in 2070 the topography of the studied region would be identical to the current topography [42]. Projection of terrestrial biome for 2070 was applied in accordance with either the contemporary situation [43] (Additional file 4, Additional file 5) or assuming modifications as in [31] (Additional file 4, Additional file 6).

### Projections of the species distribution model in future scenarios

The consensus (MaxEnt + BRT) species distribution model was projected onto two hypothetical scenarios: 1) Future scenario 1, based on the predictions by the NASA GISS-E2-R climate projection model [52] under CMIP5 RCP85 in 2070; 2) Future scenario 2, based on the predictions by the ENES HadGEM2-ES climate projection model [53] under CMIP5 RCP85 in 2070. In future scenario 1, there is an increase in temperature (2-3 °C in average) and decrease in precipitation in the driest month/quarter (−6.5-8 % mm) and in the wettest quarter (−12 % mm). Biomes and topographic features are kept unaltered. Future scenario 2 is more drastic with an increase in the highest temperature (4 °C in average), higher annual range of precipitation (i.e., wetter [+1-5 % mm in average] wettest month/quarter and drier [−15-17 % mm in average] driest month/quarter) and different biome configuration. Future scenario 1 could be considered a more conservative scenario if it was subject to less extreme events of temperature and precipitation (i.e., the coefficient of variance is smaller among predictions), while the future scenario 2 was the least optimistic as it included the possibility of extreme climate events [54], that would cause marked changes in the biome structure [31].

### Statistical analysis

Cross-validation tests between species occurrence data, absence data and each species distribution model were performed to estimate a threshold at which the sum of the sensitivity (true positive) and specificity (true negative) is highest in the confusion matrix. Evaluation of MaxEnt species distribution models was performed using background points (n = 200) as absence data (i.e., pseudo-absence). Evaluation of BRT species distribution models was done by utilizing absence data derived from MaxEnt outputs (see Table 1 for more information). The Area under the Curve (AUC) and the threshold that maximized both sensibility and specificity for each species distribution model were calculated for model evaluation.

The mean threshold for the MaxEnt and BRT species distribution models was calculated. This mean threshold value, which transformed the consensus species distribution model (probability of presence) to a binary score (presence or absence), was applied. This binary score of potential distribution of *P. falciparum* and that of each *Anopheles* species were associated in a 2 by 2 contingency table. An association metric (i.e., *odds ratio* - OR) was calculated. A result of OR < 1 showed negative association between the presence of *P. falciparum* and an *Anopheles* species and OR > 1 showed a positive association between *P. falciparum* and an *Anopheles* species. A 99 % confidence interval was adopted for the OR analysis.

## Results

**Fig. 2:**
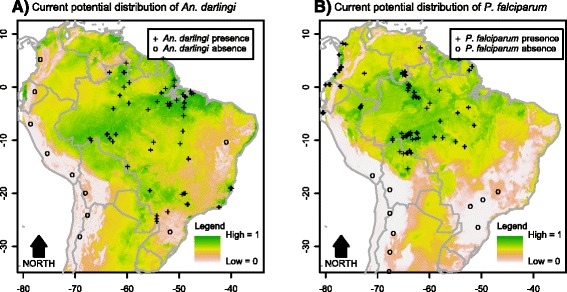
Potential distribution of *An. darlingi* and *P. falciparum* under contemporary conditions

**Fig. 3:**
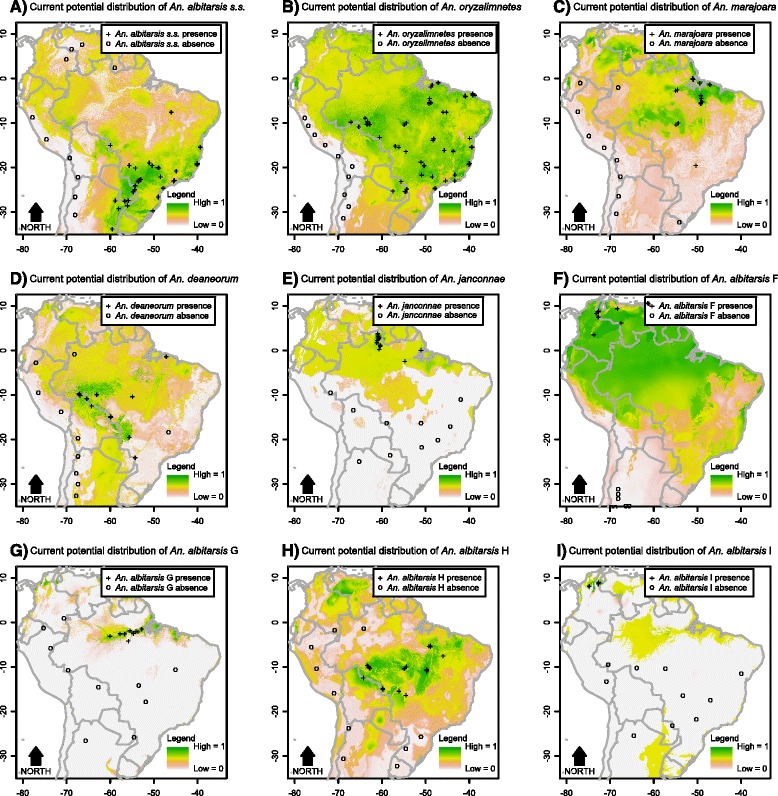
Potential distribution of members of the Albitarsis Complex under contemporary conditions

**Fig. 4:**
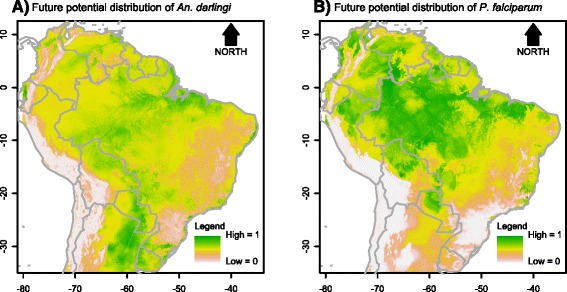
Potential distribution of *An. darlingi* and *P. falciparum* under global climate change scenario 1

**Fig. 5:**
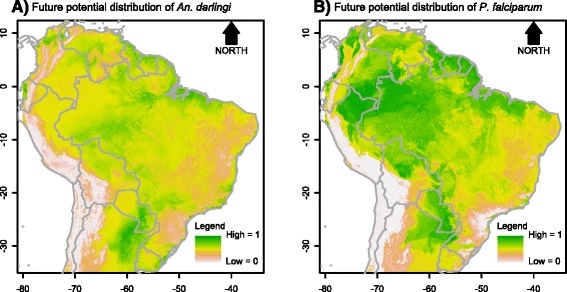
Potential distribution of *An. darlingi* and *P. falciparum* under global climate change scenario 2

**Fig. 6:**
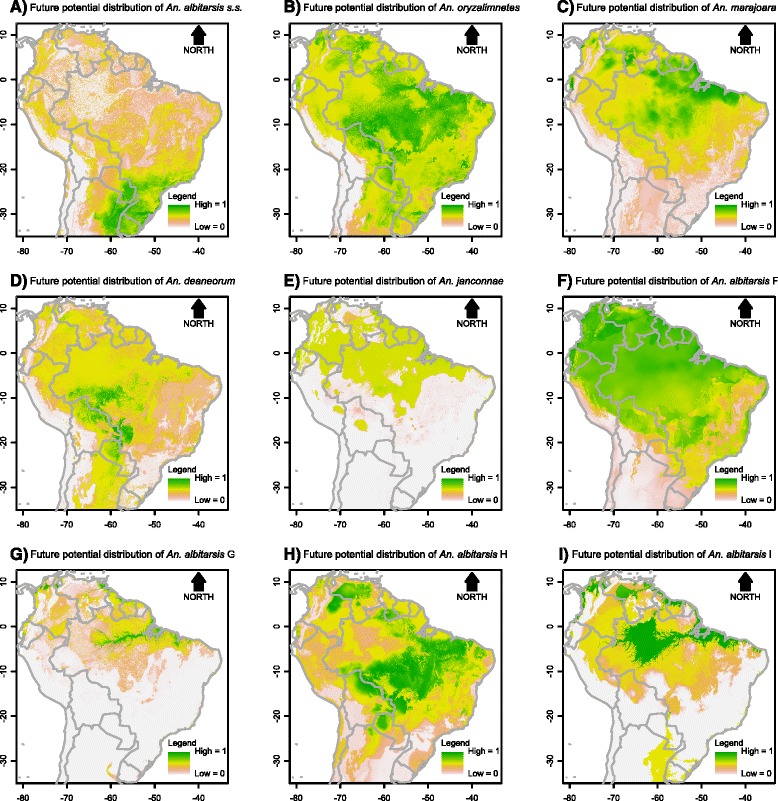
Potential distribution of members of the Albitarsis Complex under global climate change scenario 1

**Fig. 7:**
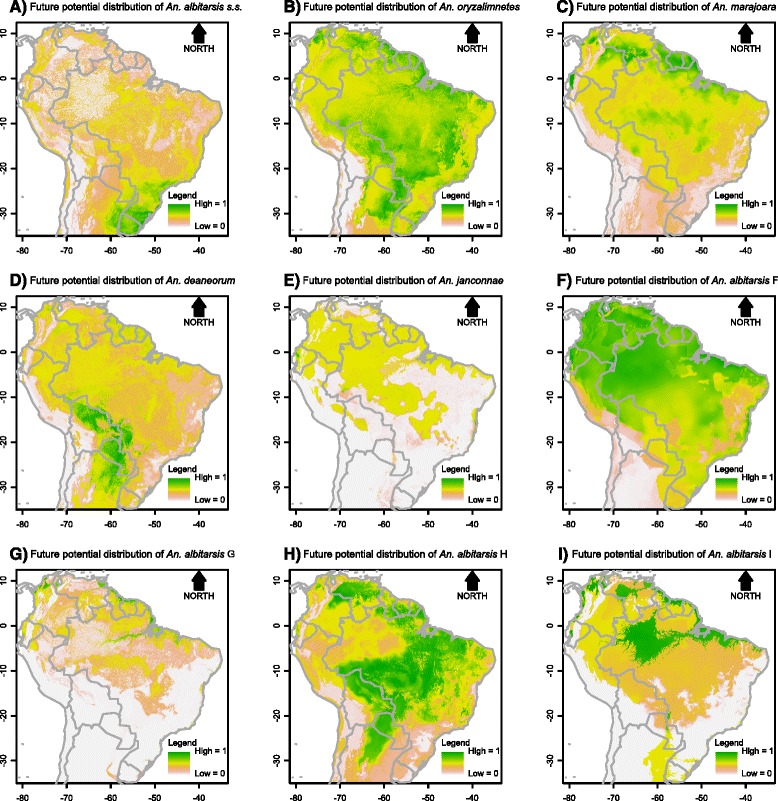
Potential distribution of members of the Albitarsis Complex under global climate change scenario 2

Contribution of each environmental variable for the habitat suitability models for the parasite, *An. darlingi* and members of the Albitarsis Complex revealed significant differential characteristics among these species, reflecting specific niche requirements (Additional file 7). In the resulting maps, the estimated probabilities of potential species presence ranged from 0 (white colour) to 1 (dark green) (see gradient legend in Figs. 2, 3, 4, 5, 6, 7).

The current spatial distribution of *Anopheles darlingi* was positively associated with warm and humid areas (Fig. 2A) and that of *P. falciparum* was positively associated with higher temperatures (Fig. 2b).

The current spatial distributions of the members of the Albitarsis Complex varied significantly (Fig. 3). As can be seen from Fig. 3, *Anopheles albitarsis* s.s. was positively associated with cold temperatures with higher potential presence in the south of the continent. *Anopheles oryzalimnetes* showed a climate-generalist characteristic with potential presence in at least three distinct biomes (Amazonia, Atlantic Forest and Brazilian Savanna). *Anopheles marajoara* was mainly associated with the drier parts of the Amazonian biome. *Anopheles deaneorum* was positively associated with dry forests and savannas. Associations of *An. janconnae* showed that this species has restricted environmental requirements with occurrence more likely in specific hotspots of the northern part of Amazonia. *Anopheles albitarsis* F was positively associated with the entire Amazonian biome. *Anopheles albitarsis* G was associated with the climatic and topographic characteristics in the Amazon River basin. *Anopheles albitarsis* H was positively associated with drier biomes such as the Brazilian Savanna. Finally, apart from the small sample size (N = 12), it was possible to extract information from *Anopheles albitarsis* I current distribution which was associated with climate and topography of the northern part of the continent.

The aforementioned habitat suitability niche-based models of *P. falciparum* and *Anopheles* species were extrapolated employing two future climate scenarios: future scenario 1, conservative [climate change only] and future scenario 2, pessimistic [climate plus biome changes].

The predicted spatial distribution maps for *An. darlingi* for both scenarios showed that this species would decrease its presence in the Amazonian biome (Figs. 4a and 5a). This could be possible because of the decrease of precipitation in both scenarios. This further shows that *An. darlingi* is not a generalist species for drier environments. On the other hand, the spatial distribution of *P. falciparum* would increase in the same biome, possibly because of the increase in temperature for both scenarios, for which this species seems to be well adapted (Figs. 4b and 5b).

The projected future distributions of species of the Albitarsis Complex are shown in Figs. 6 and 7. From such figures it can be seen that future spatial distribution of *An. albitarsis* s.s. and *An. janconnae* were restricted to the south and north, respectively, of the continent. This shows that both species are very sensitive to climate change. On the other hand, other species such as *An. deaneorum*, *An. albitarsis* F, *An. albitarsis* H, and *An. albitarsis* I showed expansion on their future spatial distribution in both scenarios, whereas *An. oryzalimnetes*, *An. marajoara*, and *An. albitarsis* G were more likely to expand their territory in future scenario 1 only. This outcome shows that some species of the Albitarsis Complex could resist climate change (*An. oryzalimnetes*, *An. marajoara*, and *An. albitarsis* G), while others may survive under climate and biome changes (*An. deaneorum*, *An. albitarsis* F, *An. albitarsis* H, and *An. albitarsis* I).

**Fig. 8:**
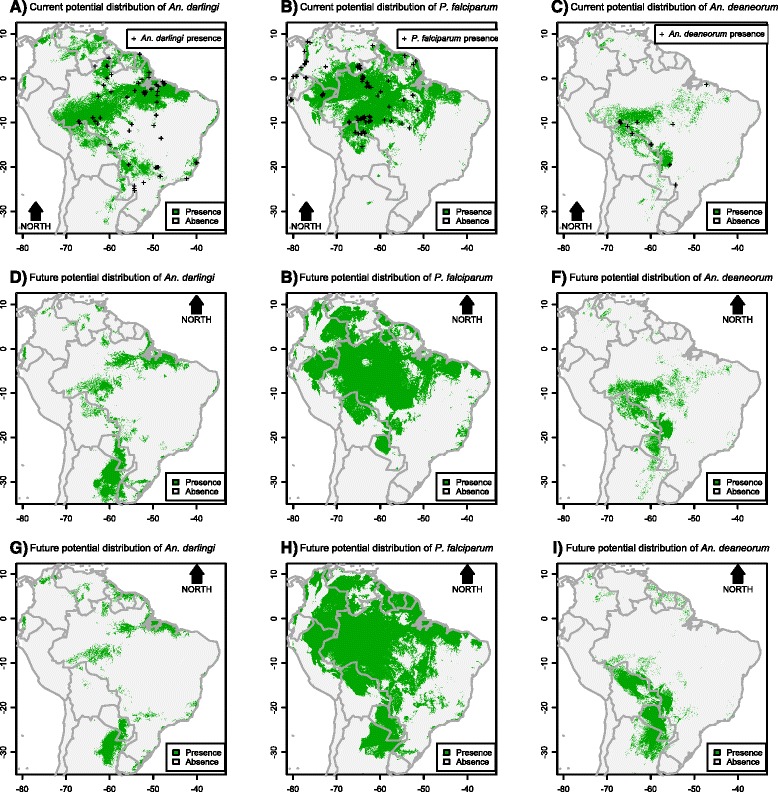
Potential distribution of *An. darlingi* and *P. falciparum* and *An. deaneorum* under contemporary conditions, global climate change scenario 1, and global climate change scenario 2

There were shifts in the predicted presence of *An. darlingi*, *P. falciparum* and *An. deaneorum* from current to future scenarios (Fig. 8). The current spatial distribution of *P. falciparum* was positively associated with higher temperatures. In future scenarios 1 and 2, in which temperature was higher, this parasite species expanded its distribution in South America. In the current scenario, coverage of *P. falciparum* in South America was 25 %, increasing to 35 % in future scenario 1 and 46 % in future scenario 2 (Fig. 8). Higher levels of precipitation positively influenced current spatial distribution of *An. darlingi*. In future scenarios 1 and 2, where precipitation decreases, its distribution in South America also decreases. The current coverage of *An. darlingi* decreased from 21 % to 11 % in scenario 1 and 8 % in scenario 2 (Fig. 8). In those Albitarsis Complex species for which precipitation was an important predictor (e.g., *An. albitarsis* s.s.), all showed decreased distributions in both future scenarios. On the contrary, other species benefitted from either temperature increase or altered biome configuration resulting in the expansion of their spatial distributions in future scenarios. For example, coverage of *An. deaneorum* increased from its current value of 6 % to 8 % in scenario 1 and 9 % in scenario 2 (Fig. 8).

Spatial associations of binary (presence/absence) species distribution models between *P. falciparum* and each *Anopheles* in all scenarios are in Table 2. Spatial association between the parasite and *An. darlingi* decreased in future scenarios. On the contrary, spatial associations between *P. falciparum* and species of the Albitarsis Complex, particularly *An. oryzalimnetes*, *An. marajoara*, *An. deaneorum*, *An. albitarsis* G, *An. albitarsis* H, and *An. albitarsis* I, increased in both future scenarios.

**Table 2:** Spatial association of species distribution models of *P. falciparum* against each *Anopheles* vector according to present and future scenarios, South America

	Current	Future (scenario1)	Future (scenario2)
	*P. falciparum*	*P. falciparum*	*P. falciparum*
	*OR* (CI99%)	*OR* (CI99%)	*OR* (CI99%)
*An. darlingi*	7.42 (7.39, 7.44)	2.48 (2.48, 2.49)	6.51 (6.47, 6.54)
*An. marajoara*	8.02 (7.97, 8.06)	9.71 (9.67, 9.77)	11.3 (11.21, 11.41)
*An. deaneorum*	6.21 (6.18, 6.24)	11.66 (11.59, 11.72)	10.25 (10.19, 10.31)
*An. janconnae*	3.41 (3.38, 3.45)	2.18 (2.14, 2.22)	0.89 (0.87, 0.91)
*An. albitarsis* s.s.	0.08 (0.08, 0.08)	0.03 (0.03, 0.03)	0.33 (0.33, 0.33)
*An. oryzalimnetes*	1.91 (1.9, 1.91)	9.21 (9.18, 9.24)	3.13 (3.12, 3.14)
*An. albitarsis *F	10.29 (10.26, 10.33)	5.41 (5.39, 5.42)	6.92 (6.9, 6.94)
*An. albitarsis *G	4.72 (4.66, 4.77)	7.55 (7.46, 7.64)	28.83 (27.78, 29.89)
*An. albitarsis *H	3.48 (3.47, 3.49)	9.36 (9.33, 9.39)	6.57 (6.55, 6.58)
*An. albitarsis *I	1.2 (1.16, 1.23)	21.6 (21.48, 21.73)	44.89 (44.38, 45.34)

## Discussion

Probable spatial distributions of the malarial parasite *P. falciparum* and ten Neotropical *Anopheles* species were predicted using an ecological niche modelling approach that included climate factors, topographical and biomic variables under the current malaria transmission setting in South America. In a second round of analyses, the currently predicted niche model for each species was addressed employing two potential scenarios of future climate and biome changes in 2070, with the assumption that climate change would progress under a high emission scenario (i.e., RCP8.5). A significant potential shift in the respective roles of the current major mosquito vector species of *P. falciparum* was indicated using two distinct scenarios of climate and environmental changes predicted for 2070. Our analyses showed that whereas the role of the major current vector *An. darlingi* in malaria transmission may decrease in future, some species of the Albitarsis Complex (*An. marajoara*, *An. deaneorum*, *An. albitarsis* G, *An. albitarsis* H, and *An. albitarsis* I) may adopt a more significant role as either primary or secondary vectors of *P. falciparum* in South America. Because of a potential distribution expansion in some species of the Albitarsis Complex, the geographical distribution of the parasite could exacerbate, from few-clustered parasite populations at present to a hugely increased potential 35-46 % coverage of South America by 2070. This result is consistent with the results found by Caminade et al. [11], in which they showed that *P. falciparum* would expand its current distribution towards areas situated in greater latitudes and altitudes, if the temperatures in those areas increased. Additional supporting evidences were found in highland ecosystems in where parasites and vectors had expanded their geographic distributions towards malaria-free highlands because of climatic and socio-economic changes [55, 56].

The distribution of *An. darlingi,* which is dependent on high precipitation levels [57], decrease markedly in both future scenarios of climate changes tested in the present study. This result is consistent with the arguments of Parham and Michael [10] regarding the association of climate variables, such as levels of precipitation and temperature, and their role as potential driving forces to the rate of malaria transmission. This further relates to the evidences that an optimum threshold of malaria emergence could occur under combining effects of temperature and precipitation [58, 59]. In contrast, spatial distributions of the component species of the Albitarsis Complex seem to be less dependent on precipitation levels [24]. The evidence that effects of climate change on vector abundance require species-specific combinations further supports this result [60]. Therefore, in future scenarios subject to climate changes, dynamics of malaria could have such species, particularly *An. marajoara* and *An. deaneorum*, as local primary vectors of *P. falciparum* in forest areas that can follow a savannization process.

The core message in the present work is that future scenarios may lead to shifts in the relative importance of species of the Albitarsis Complex over *An. darlingi* for transmission of *P. falciparum* in South America. It is not possible, however, at this stage, to confirm that future distribution of malaria will decrease or not because the human control efforts were not included as predictors in the projected scenarios. Additionally, further implementations on the projected scenarios would be to consider the effects of future climate change on the quality of larval habitat, as this potentially is an important predictor to malaria transmission [61]. On the other hand, Foley et al. [24], who utilized the same dataset of specimens of the Albitarsis Complex used herein, but in a different approach, showed that vector species shifts are likely to occur in South America. More specifically, these authors showed that long term changes in precipitation could affect vector abundance and distribution differently, possibly increasing the chances of *An. marajoara* compared to species more sensitive to water unavailability, such as *An. albitarsis* s.s., or *An. darlingi*, according to the present study. Vector species shifts may further relate to the magnitude of vector richness in a certain region because the pool of competent vectors in a mosquito assemblage could likely be an important predictor to malaria resilience, as supported recently [62].

The link between future scenarios under climate change effects and Amazonian savannization process has long been recognized [31, 32]. During a savannization process, Amazonian tropical evergreen forests would be replaced by drier and less productive biomes such as savanna, shrub land or even semi-desert [31]. Amazonian forest could be subject to a decreased precipitation, and a few models suggest the extensive possibility of retreat of the forest [32]. Potential interactions between climate changes and land-use changes may strongly influence the Amazonian ecosystem in the future [28]. Changes in land-use and human migration patterns have already affected malaria transmission in areas of the municipality of Macapá, in Amapá state in the Amazon forest [26], with the replacement of the primary vector *An. darlingi* by a secondary vector *An. marajoara* [26]. The ecological niches of members of the Albitarsis Complex are dependent upon long-term changes in precipitation and, especially, the increased duration of the dry season can increase the abundance and distribution of some species of this group [24]. This may be indicative that these species will succeed at colonizing savanna forest in future scenarios.

## Conclusions

Climatic and landscape effects caused by global warming will facilitate expanded distributions and, consequentially, increasingly important roles for component species in the Albitarsis Complex as important malaria vectors. This further highlights the need for further detailed studies elucidating the respective bionomics, ecology and epidemiological roles of component members of the Albitarsis Complex.

## Additional files

Additional file 1:
**Tabular data in Excel Spreadsheet.** *Anopheles darlingi* Occurrence Data. (XLSX 12 kb) Additional file 2: Figure S9. Environmental predictors (bioclimatic data, elevation and terrestrial biomes) under contemporary conditions. Sources: Hijmans et al. [41], the Shuttle Radar Topography Mission [42] and the World Wildlife Fund [43]. (PDF 292 kb) Additional file 3: Table S3. Description of environmental variables utilized in the niche modelling of *P. falciparum* and *Anopheles* vectors in South America, at present. (DOCX 14 kb) Additional file 4: Table S4. Description of environmental variables utilized in simulations of future scenarios of the niche model of *P. falciparum* and *Anopheles* vectors in South America, 2070. (DOCX 15 kb) Additional file 5: Figure S10. Environmental variables (bioclimatic data, topographic and terrestrial biomes) under global climate change scenario 1. Sources: the NASA Goddard Institute for Space Studies [52], the Shuttle Radar Topography Mission [42] and the World Wildlife Fund [43]. (PDF 293 kb) Additional file 6: Figure S11. Environmental variables (bioclimatic data, topographic and terrestrial biomes) under global climate change scenario 2. Sources: the ENES Met Office Hadley Centre [53], the Shuttle Radar Topography Mission [42] and Lapola et al. [31]. (PDF 321 kb) Additional file 7: Table S5. Relative contribution (%) of environmental variables to predicted values in the MaxEnt algorithm and Boosted Regression Trees (BRT) for each species in South America. (DOCX 16 kb)
