# Supplementary figures and images for: Malaria vectors in South America: current and future scenarios
Source: Parasit Vectors. 2015 Aug 19;8:426. doi: 10.1186/s13071-015-1038-4 (PMC4539674; doi:10.1186/s13071-015-1038-4)

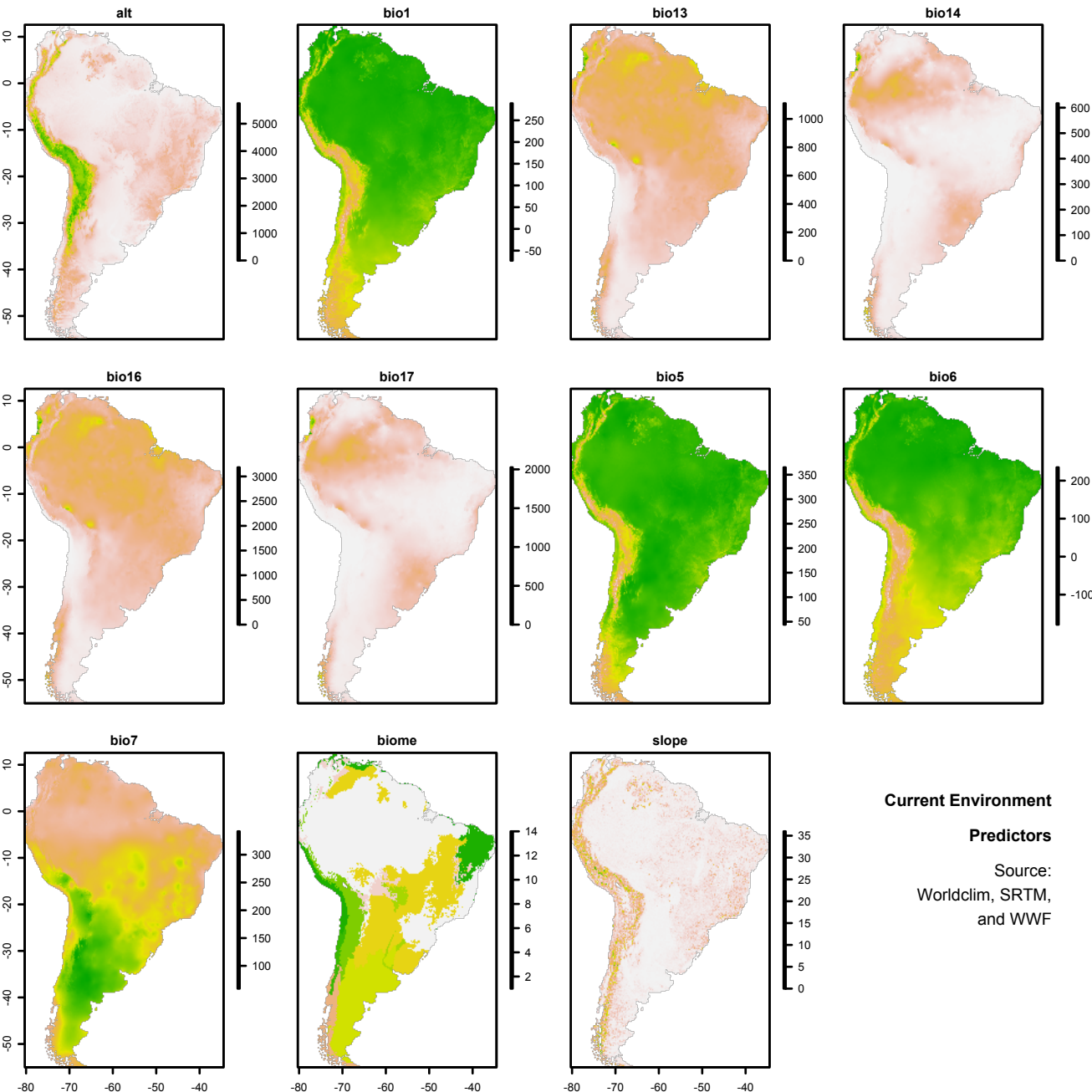

Supplement: Additional file 2: Figure S9. — Environmental predictors (bioclimatic data, elevation and terrestrial biomes) under contemporary conditions. Sources: Hijmans et al. [41], the Shuttle Radar Topography Mission [42] and the World Wildlife Fund [43]. (PDF 292 kb) [file 13071_2015_1038_MOESM2_ESM.pdf]

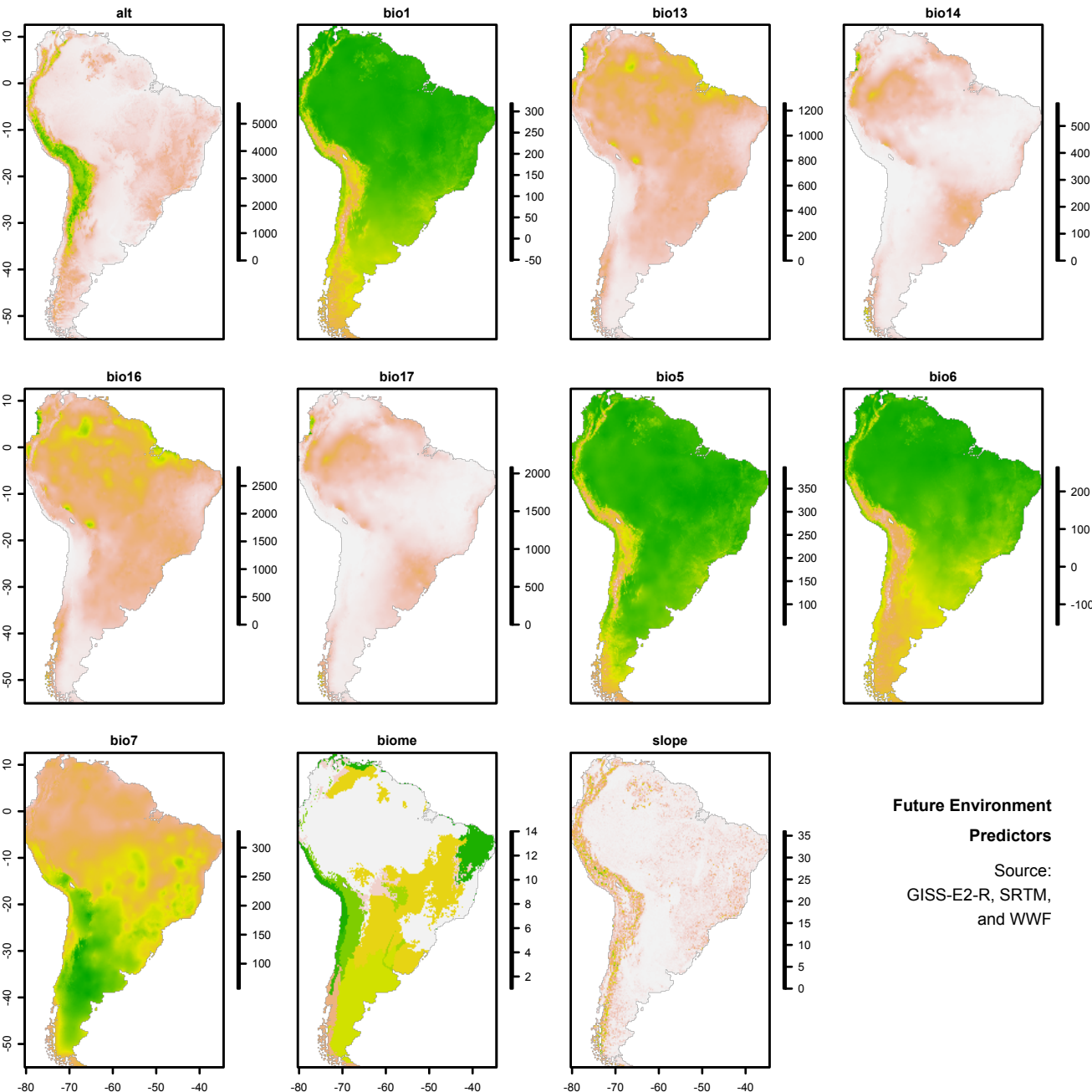

Supplement: Additional file 5: Figure S10. — Environmental variables (bioclimatic data, topographic and terrestrial biomes) under global climate change scenario 1. Sources: the NASA Goddard Institute for Space Studies [52], the Shuttle Radar Topography Mission [42] and the World Wildlife Fund [43]. (PDF 293 kb) [file 13071_2015_1038_MOESM5_ESM.pdf]

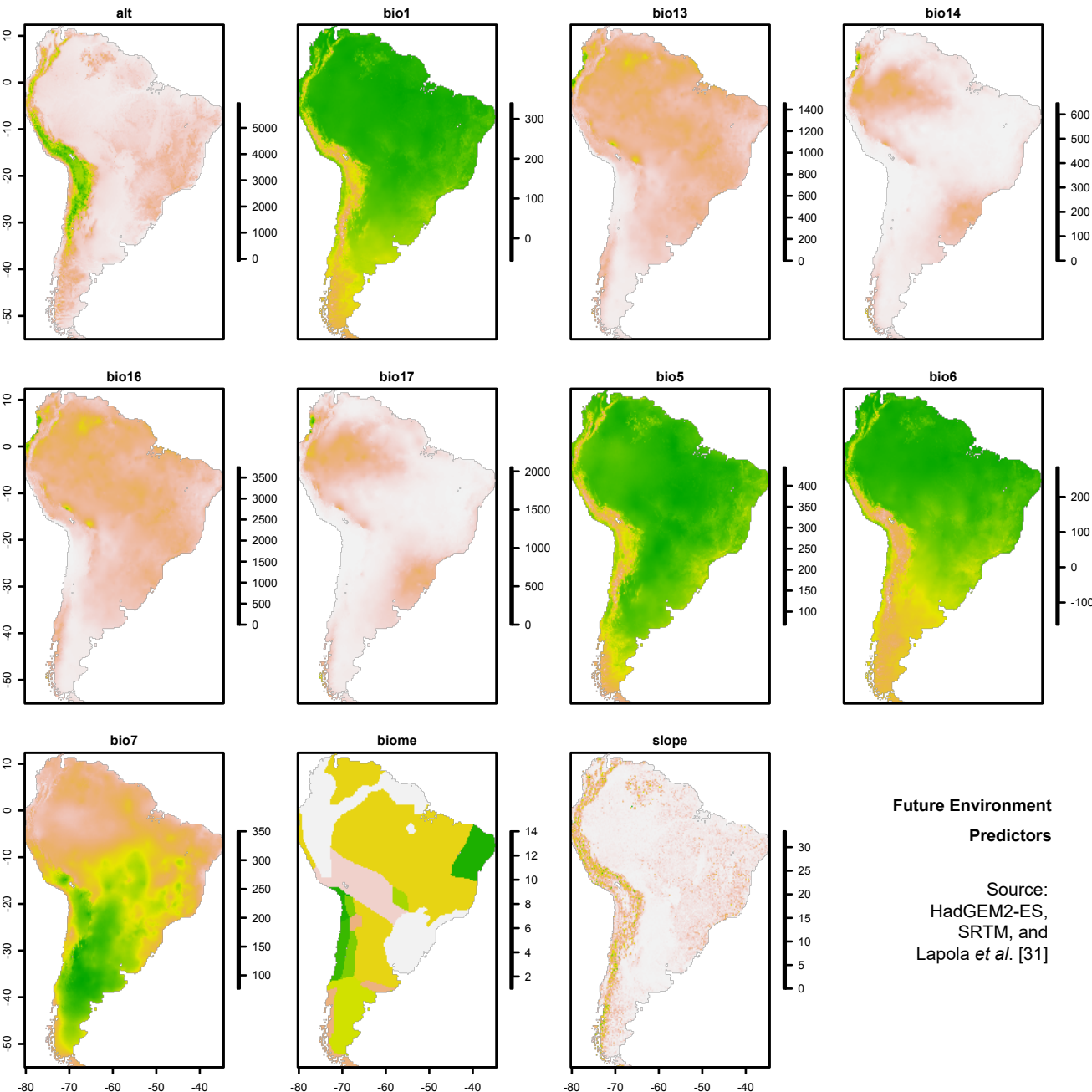

Supplement: Additional file 6: Figure S11. — Environmental variables (bioclimatic data, topographic and terrestrial biomes) under global climate change scenario 2. Sources: the ENES Met Office Hadley Centre [53], the Shuttle Radar Topography Mission [42] and Lapola et al. [31]. (PDF 321 kb) [file 13071_2015_1038_MOESM6_ESM.pdf]
