# Supplementary material for: Malaria vectors in South America: current and future scenarios
Source: Parasit Vectors. 2015 Aug 19;8:426. doi: 10.1186/s13071-015-1038-4 (PMC4539674; doi:10.1186/s13071-015-1038-4)
Supplement: Additional file 3: Table S3. — Description of environmental variables utilized in the niche modelling of P. falciparum and Anopheles vectors in South America, at present. (DOCX 14 kb) [file 13071_2015_1038_MOESM3_ESM.docx]

**Additional file 3: Table S3 Description of environmental variables utilized in the niche modelling of *P*. *falciparum* and *Anopheles* vectors in South America, at present**

| **Environmental variables^a^** | **Summary^b^** | **Units** | **Sources** |
| --- | --- | --- | --- |
| Annual Mean Temperature (BIO1) | 21.2 (6.6), ˗15.4-29.2 | °C | WorldClim |
| Maximum Temperature of the Warmest Month (BIO5) | 30.1 (5.4), ˗3.3-36.6 | °C | WorldClim |
| Minimum Temperature of the Coldest Month (BIO6) | 12.3 (8.6), ˗26-24.1 | °C | WorldClim |
| Temperature Annual Range - BIO5 minus BIO6 (BIO7) | 17.7 (5.8), 5.7-34.3 | °C | WorldClim |
| Precipitation of Wettest Month (BIO13) | 228 (122.2), 0-1325 | mm | WorldClim |
| Precipitation of Driest Month (BIO14) | 40.9 (49.8), 0-752 | mm | WorldClim |
| Precipitation of Wettest Quarter (BIO16) | 624.8 (341.2), 0-3794 | mm | WorldClim |
| Precipitation of Driest Quarter (BIO17) | 146.3 (163.2), 0-2495 | mm | WorldClim |
| Elevation database (ALT) | 590 (926), ˗256-6740 | meters | SRTM |
| Slope Terrain (SLOPE)^c^ | 1.8 (3.4), 0-42.6 | ° | SRTM |
| Terrestrial biomes and eco-regions (BIOME) | - | categorical | WWF |

^a^ Spatial resolution: 1-km (30 arc-seconds). Projection: longitude-latitude. Datum: WGS84.

^b^ Descriptive summary of a continuous variable: mean (standard deviation), min.-max..

^c^ Derived from elevation database.
