# Supplementary material for: Malaria vectors in South America: current and future scenarios
Source: Parasit Vectors. 2015 Aug 19;8:426. doi: 10.1186/s13071-015-1038-4 (PMC4539674; doi:10.1186/s13071-015-1038-4)
Supplement: Additional file 4: Table S4. — Description of environmental variables utilized in simulations of future scenarios of the niche model of P. falciparum and Anopheles vectors in South America, 2070. (DOCX 15 kb) [file 13071_2015_1038_MOESM4_ESM.docx]

**Additional file 4: Table S4** **Description of environmental variables utilized in simulations of future scenarios of the niche model of *P*. *falciparum* and *Anopheles* vectors in South America, 2070**

| **Environmental variables^a^** | **Summary^b^** | **Units** | **Sources^c^** |
| --- | --- | --- | --- |
| BIO1 | 23.8 (6.9), ˗13.2-32 | °C | GISS-E2-R |
|  | 25.5 (7.1), ˗11.8-34 | °C | HadGEM2-ES |
| BIO5 | 32.8 (5.5), ˗2.1-39.9 | °C | GISS-E2-R |
|  | 35.8 (6.2), 7-44.6 | °C | HadGEM2-ES |
| BIO6 | 15 (8.7), ˗23.6-26.4 | °C | GISS-E2-R |
|  | 16.3 (9.1), ˗22.6-28.5 | °C | HadGEM2-ES |
| BIO7 | 17.8 (5.2), 5.3-33.3 | °C | GISS-E2-R |
|  | 19.5 (5.9), 6.4-35.1 | °C | HadGEM2-ES |
| BIO13 | 229.2 (130.6), 0-1364 | mm | GISS-E2-R |
|  | 239.4 (132.7), 0-1521 | mm | HadGEM2-ES |
| BIO14 | 37 (44), 0-736 | mm | GISS-E2-R |
|  | 34.3 (47.3), 0-690 | mm | HadGEM2-ES |
| BIO16 | 616.6 (350.4), 0-3131 | mm | GISS-E2-R |
|  | 628 (347.8), 0-3937 | mm | HadGEM2-ES |
| BIO17 | 135.2 (149.3), 0-2575 | mm | GISS-E2-R |
|  | 127.6 (160.2), 0-2213 | mm | HadGEM2-ES |
| ALT | 590 (926), ˗256-6740 | meters | SRTM |
| SLOPE^d^ | 1.8 (3.4), 0-42.6 | ° | SRTM |
| BIOME | - | categorical | WWF |
|  | - | categorical | Lapola *et al*. [31]^e^ |

^a^ Spatial resolution: 1-km (30 arc-seconds). Projection: longitude-latitude. Datum: WGS84.

^b^ Descriptive summary of a continuous variable: mean (standard deviation), min.-max.

^c^ GISS-E2-R: NASA Goddard Institute for Space Studies’ climate projection model to the Coupled Model Intercomparison Project Phase 5 (CMIP5), under the RCP85 (+8.5W/m^2^). HadGEM2-ES: ENES Met Office Hadley Centre’s climate projection model to the CMIP5, under the RCP85 (+8.5W/m^2^).

^d^ Derived from elevation database.

^e^ WWF’s BIOME modified according to Lapola *et al*. [31]. Modifications were based on the authors’ predictions using ENES Met Office Hadley Centre’s climate projection model that large portions of Amazonia is replaced by tropical savanna in 2070-2099.
