# Supplementary material for: Malaria vectors in South America: current and future scenarios
Source: Parasit Vectors. 2015 Aug 19;8:426. doi: 10.1186/s13071-015-1038-4 (PMC4539674; doi:10.1186/s13071-015-1038-4)
Supplement: Additional file 7: Table S5. — Relative contribution (%) of environmental variables to predicted values in the MaxEnt algorithm and Boosted Regression Trees (BRT) for each species in South America. (DOCX 16 kb) [file 13071_2015_1038_MOESM7_ESM.docx]

**Additional file 7: Table S5 Relative contribution (%) of environmental variables to predicted values in the MaxEnt algorithm and Boosted Regression Trees (BRT) for each species in South America**

|  |  | BIO1 | BIO5 | BIO6 | BIO7 | BIO13 | BIO14 | BIO16 | BIO17 | ALT | SLOPE | BIOME |
| --- | --- | --- | --- | --- | --- | --- | --- | --- | --- | --- | --- | --- |
| *P. falciparum* | Maxent | 21.7 | 8.8 | 12.1 | 1.4 | 4.8 | 1.2 | 5.4 | 12.9 | 25.5 | 1.7 | 4.5 |
|  | BRT | 6.6 | 21 | 11.1 | 12.2 | 11.5 | 0.2 | 11.4 | 1 | 23.6 | 0.6 | 0.8 |
| *An. darlingi* | Maxent | 3.3 | 3.2 | 2.2 | 0.04 | 0 | 14.9 | 29.2 | 7.3 | 28.5 | 9.1 | 2.3 |
|  | BRT | 8.4 | 6.4 | 4.8 | 0.8 | 9.9 | 2.7 | 5.1 | 3.1 | 37.2 | 21.4 | 0.3 |
| *An. albitarsis* s.s. | Maxent | 8.7 | 2 | 27.5 | 2.3 | 5.6 | 15.9 | 9.7 | 0 | 11.1 | 11.7 | 5.5 |
|  | BRT | 8.5 | 10.5 | 0.7 | 9.1 | 2.5 | 30.1 | 2.6 | 4.3 | 15.7 | 13.5 | 2.5 |
| *An. oryzalimnetes* | Maxent | 2.3 | 6.9 | 17.7 | 1 | 0.1 | 2.9 | 16.4 | 12.9 | 11.3 | 23.2 | 5.3 |
|  | BRT | 17.8 | 2 | 30.9 | 1.2 | 21.7 | 0 | 0.7 | 0.9 | 20 | 2.7 | 2 |
| *An. marajoara* | Maxent | 1.6 | 0 | 23 | 0 | 19.1 | 3.6 | 6.7 | 28 | 4 | 0 | 14 |
|  | BRT | 46.6 | 10.4 | 10.6 | 4.5 | 7.8 | 0.4 | 3.2 | 0.5 | 2.4 | 8.1 | 5.6 |
| *An. deaneorum* | Maxent | 0.1 | 0.6 | 13.2 | 0 | 12.9 | 6.4 | 7 | 5.5 | 10.1 | 27.7 | 16.5 |
|  | BRT | 2.7 | 17.8 | 0.5 | 0.2 | 6.2 | 1 | 6.4 | 2.3 | 18.3 | 40.7 | 3.8 |
| *An. janconnae* | Maxent | 17.3 | 6.9 | 29.9 | 0.5 | 2.6 | 5.7 | 0.1 | 4.4 | 8.4 | 1.1 | 23.1 |
|  | BRT | 12.5 | 1.3 | 1.5 | 21.9 | 13.5 | 2 | 20.8 | 0.2 | 24.5 | 1.8 | 0 |
| *An. albitarsis* F | Maxent | 0 | 0 | 0 | 88.1 | 0 | 0 | 0 | 0 | 2.7 | 0 | 9.3 |
|  | BRT | 26.2 | 0.3 | 5.9 | 10.7 | 15.3 | 10.6 | 2.2 | 1.6 | 9.6 | 0.1 | 17.5 |
| *An. albitarsis* G | Maxent | 0 | 0.1 | 51.5 | 0 | 1.5 | 1.8 | 0 | 7.3 | 27.9 | 0.6 | 9.4 |
|  | BRT | 18.3 | 0.3 | 14.2 | 21.7 | 1.5 | 0.8 | 0 | 0.7 | 40 | 2.3 | 0 |
| *An. albitarsis* H | Maxent | 3.5 | 10 | 6.6 | 0.01 | 0 | 25.5 | 22.1 | 9.8 | 9.6 | 10.9 | 2 |
|  | BRT | 1.4 | 21.1 | 1.6 | 16.7 | 18.7 | 4.8 | 15.3 | 10.2 | 6.5 | 3.2 | 0.5 |
| *An. albitarsis* I | Maxent | 91.7 | 0 | 0 | 0 | 1.1 | 1.3 | 0 | 0 | 0 | 0.2 | 5.6 |
|  | BRT | 12.7 | 0 | 0 | 0 | 0 | 0 | 0 | 0 | 87.3 | 0 | 0 |
